# Genome-Based Targeted Sequencing as a Reproducible Microbial Community Profiling Assay

**DOI:** 10.1128/mSphere.01325-20

**Published:** 2021-04-07

**Authors:** Jacquelynn Benjamino, Benjamin Leopold, Daniel Phillips, Mark D. Adams

**Affiliations:** a The Jackson Laboratory for Genomic Medicine, Farmington, Connecticut, USA; Baylor College of Medicine

**Keywords:** 16S, MA-GenTA, mWGS, microbial communities, microbiome, targeted sequencing

## Abstract

Current sequencing-based methods for profiling microbial communities rely on marker gene (e.g., 16S rRNA) or metagenome shotgun sequencing (mWGS) analysis. We present an approach based on a single-primer extension reaction using a highly multiplexed oligonucleotide probe pool. This approach, termed MA-GenTA (microbial abundances from genome tagged analysis), enables quantitative, straightforward, cost-effective microbiome profiling that combines desirable features of both 16S rRNA and mWGS strategies. The use of multiple probes per target genome and rigorous probe design criteria enabled robust determination of relative abundance. To test the utility of the MA-GenTA assay, probes were designed for 830 genome sequences representing bacteria present in mouse stool specimens. Comparison of the MA-GenTA data with mWGS data demonstrated excellent correlation down to 0.01% relative abundance and a similar number of organisms detected per sample. Despite the incompleteness of the reference database, nonmetric multidimensional scaling (NMDS) clustering based on the Bray-Curtis dissimilarity metric of sample groups was consistent between MA-GenTA, mWGS, and 16S rRNA data sets. MA-GenTA represents a potentially useful new method for microbiome community profiling based on reference genomes.

**IMPORTANCE** New methods for profiling the microbial communities can create new approaches to understanding the composition and function of those communities. In this study, we combined bacterial genome-specific probe design with a highly multiplexed single primer extension reaction as a new method to profile microbial communities, using stool from various mouse strains as a test case. This method, termed MA-GenTA, was benchmarked against 16S rRNA gene sequencing and metagenome sequencing methods and delivered similar relative abundance and clustering data. Since the probes were generated from reference genomes, MA-GenTA was also able to provide functional pathway data for the stool microbiome in the assayed samples. The method is more informative than 16S rRNA analysis while being less costly than metagenome shotgun sequencing.

## INTRODUCTION

The primary molecular methods for determining microbial composition are based on marker gene sequencing or whole-metagenome shotgun sequencing (mWGS). The 16S rRNA marker gene has been widely used for bacterial profiling for decades across diverse ecosystems (1, 2). Using this method, taxonomic classification of the bacterial community can be obtained at modest cost and a resolution that ranges from subspecies to family level, depending on the 16S rRNA segment that is sequenced (4–6). Continued reduction in the cost of DNA sequencing has meant that mWGS approaches have become increasingly common due to the greater information on gene content, taxonomic resolution, and strain-level variation (7), despite higher cost and complexity of data analysis.

The Human Microbiome Project (8) and similar large-scale investments (9) established methods and reference data sets for characterization of microbial profiles across diverse human body sites. As a result, the tools and reference genome data sets for characterizing human microbiomes are much better developed than those involving other organisms. The mouse is widely used in microbiome studies that seek to demonstrate a causal role of microbes affecting a given trait and to understand the mechanisms by which microbes contribute to phenotypes (10). The vast majority of mWGS sequences from mouse gut samples have no matches to named organisms in public databases (11), substantially limiting the informativeness of this approach.

One approach to overcome the limited availability of reference genome sequences is construction of *in silico* genomes based on computational sequence assembly of large mWGS data sets to create metagenome-assembled genomes (MAGs) (12–14). The integrated Mouse Gut Metagenomic Catalog (iMGMC) (15) is one such effort. Combining 1.3 Tbp of data from 298 mouse metagenomic libraries, Lesker et al. (15) assembled 1.2 million contigs; a subset of these could be grouped into 830 high-quality MAGs (hqMAGs) that are predicted to be >90% complete and <5% contaminated based on the representation of single-copy genes (16).

**FIG 1:**
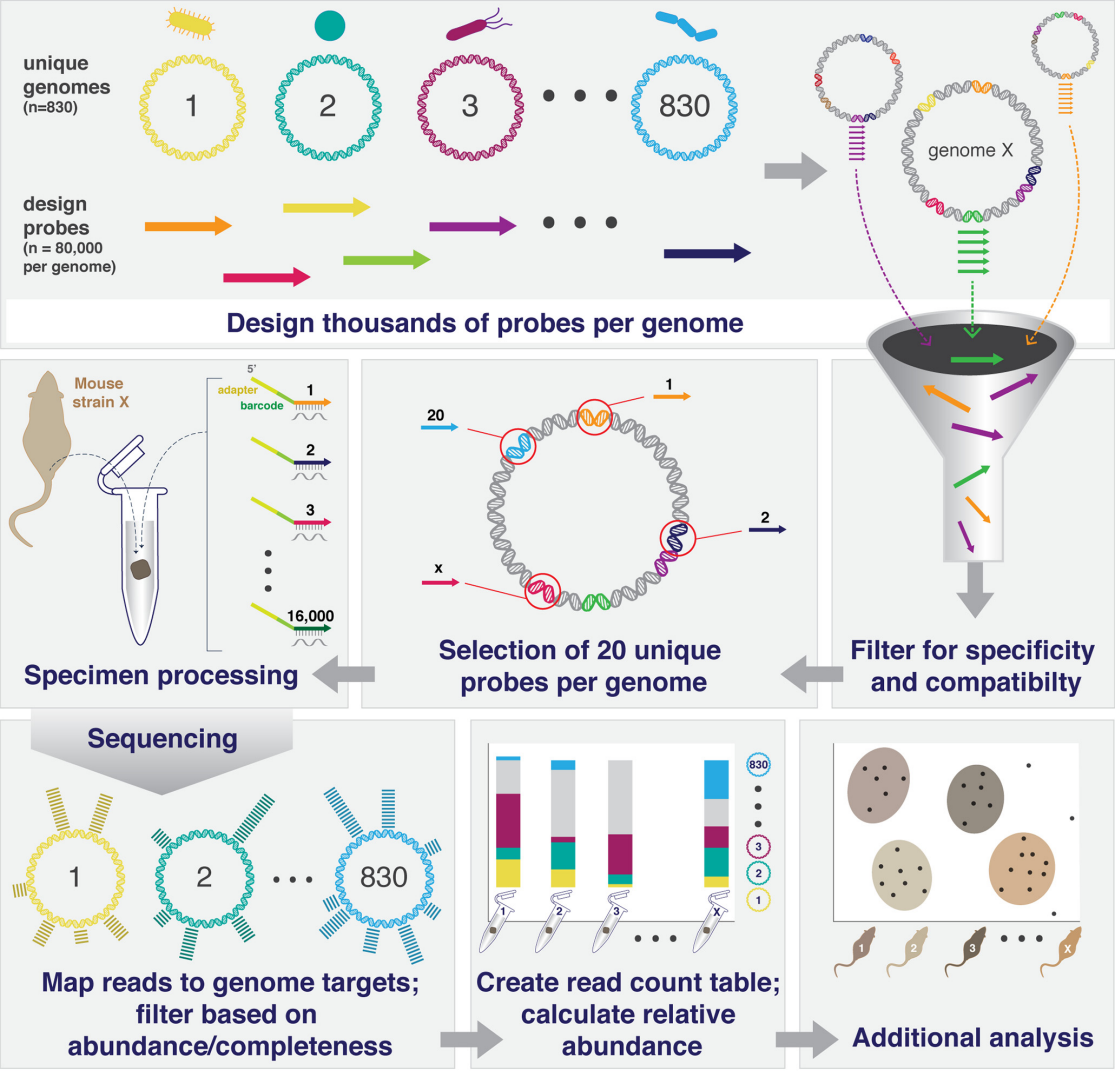
Overview of the MA-GenTA strategy. MA-GenTA utilizes software (CATCH) to design thousands of probes per genome for multiple genomes (830 in this study). All probes from the initial design are filtered based on multiple parameters (% GC, BLAST matches to inclusion/exclusion lists, nonunique matches across genomes, etc.). Unique probes are selected for each genome (20 in this study). Probe pools are synthesized and used to prepare sequencing libraries using the Allegro Targeted Genotyping kit and then sequenced. Reads are then mapped to the reference genomes to produce count tables for downstream analysis.

Here, we describe a new approach to metagenome profiling termed MA-GenTA (Microbial Abundances from Genome Tagged Analysis) that combines the specificity of mWGS analysis with a simplified laboratory and analytical workflow (Fig. 1). The availability of custom-designed highly multiplexed pools of oligonucleotides has opened possibilities for a range of new assay methods to specifically target microbes at the species, strain, and even gene level. We adapted the Allegro Targeted Genotyping assay’s single primer extension reaction that is widely used for genotyping (17, 18) and implemented it as a quantitative, straightforward, and cost-effective method for profiling mouse microbial communities based on the iMGMC hqMAGs.

## RESULTS

The MA-GenTA assay is based on approximating the relative abundance of hundreds of microbial species using sets of probes designed to be unique to each genome. The approach includes design of compatible probes directed at the genomes (or genes) of interest, library construction that uses the probe pools in a primer extension reaction, and integration of data across multiple probes to determine species abundance (Fig. 1). Oligonucleotide probe sets were designed representing 830 iMGMC hqMAGs (15). The iMGMC hqMAGs are currently the most comprehensive genome reference available for mouse gut microbes, and on average, 50% of metagenome reads from mouse stool samples analyzed here mapped to the hqMAGs (see Table S1 in the supplemental material).

10.1128/mSphere.01325-20.6 TABLE S1 Read counts and mapping statistics. Download Table S1, XLSX file, 0.02 MB. Copyright © 2021 Benjamino et al. 2021 Benjamino et al. https://creativecommons.org/licenses/by/4.0/ This content is distributed under the terms of the Creative Commons Attribution 4.0 International license.

Computational analysis suggested that each hqMAG is consistent with representing a single bacterial species, and about 12% of hqMAGs are concordant with genome sequences of bacterial isolates that are present in GenBank (15). Most hqMAGs, though, do not correspond with isolated bacteria or finished-quality genome sequences, so in considering a probe design strategy, we decided to develop two completely independent sets of 20 probes for each hqMAG. We reasoned that concordance of relative abundance between these probe sets would provide additional support for the conjecture that the hqMAGs are reasonable approximations of bona fide genome sequences and that the organisms they represent are commonly found in the mouse gut.

Two defined-composition genomic DNA positive controls and a no-template negative control (NTC) were initially used to assess the specificity of each probe set. Escherichia coli genomic DNA (gDNA) and the ZymoBIOMICS Microbial Community Standard (Mock), which contains three species present in the iMGMC hqMAG set, one of which is an E. coli strain, were used as the positive controls.

**FIG 2:**
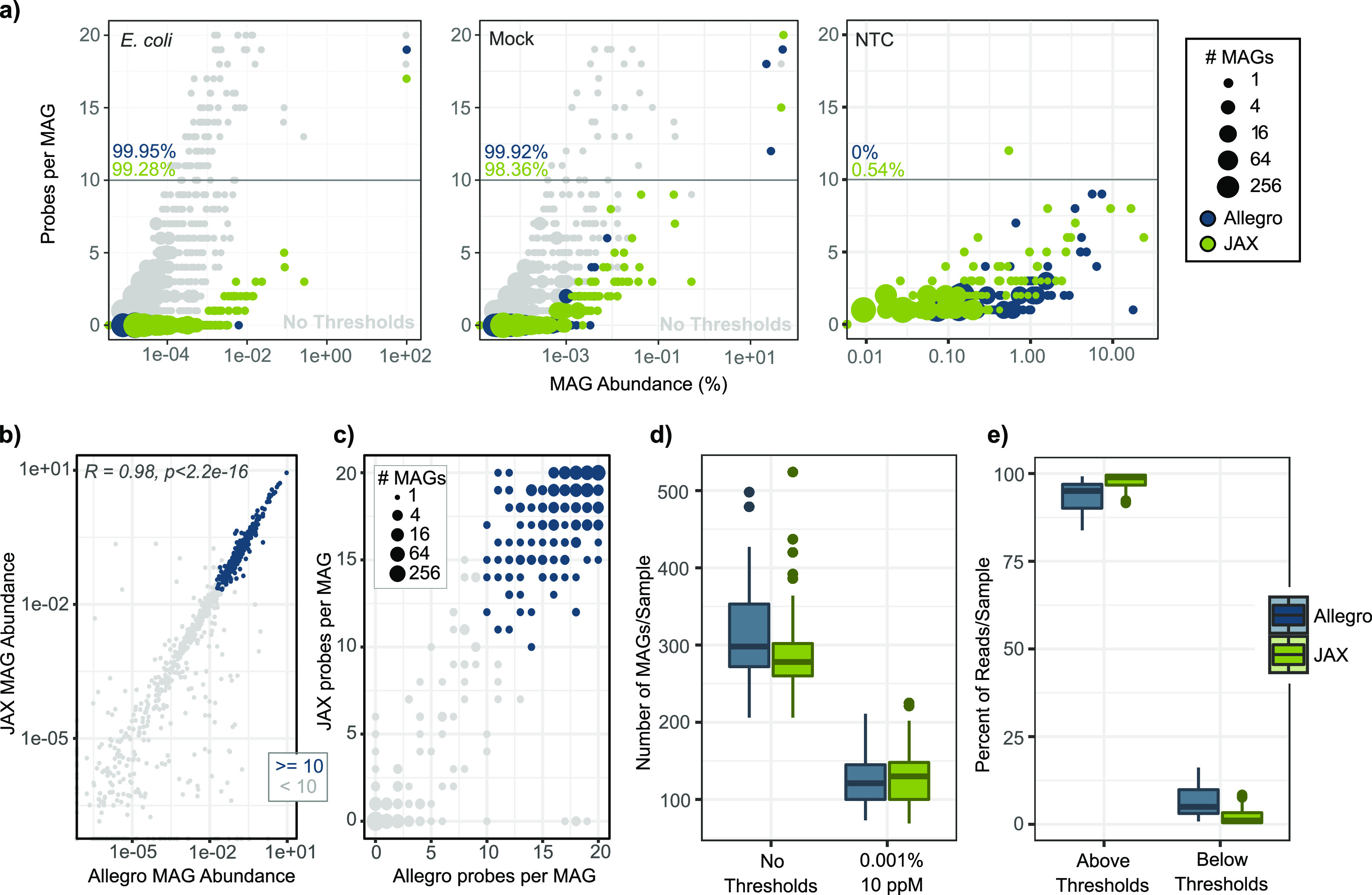
Use of control samples to establish thresholds for defining MAG presence. Thresholds for declaring a MAG present in a sample were determined using Escherichia coli genomic DNA, ZymoBIOMICS Microbial Community Standard, and a no-template control (NTC). (a) The number of probes present for each MAG (*y* axis) and the MAG abundance (*x* axis) for each control sample before applied thresholds are shown in gray. Blue (Allegro) and green (JAX) points indicate MAGs detected in each control sample when considering only probes with ≥0.001% abundance. (b) The percent relative abundance of each MAG in each sample based on the Allegro design (*x* axis) and the JAX design (*y* axis) is shown. MAGs with 10 or more probes above the 0.001% probe-abundance threshold in both designs are shown in blue. Pearson correlation of the two designs is *R* = 0.98. (c) The number of probes per MAG detected using the Allegro design (*x* axis) and JAX design (*y* axis). As in panel b, MAGs with at least 10 probes with ≥0.001% abundance in both assays are colored blue. Most MAGs have ≥10 probes per MAG above the threshold (top right) or ≤5 (bottom left). (d) The number of MAGs detected per sample with minimum probe abundance and probe representation (probes per MAG [ppM]) thresholds is shown compared to the number of MAGs detected with no thresholds across mouse samples. (e) Percentage of reads that map to MAGs with and without the probe representation thresholds.

Alignment of primary sequence reads showed that probes from many MAGs were detected for the Allegro and JAX designs for E. coli and Mock samples (gray dots in Fig. 2a), the vast majority represented by a small number of probes with low relative abundance (Fig. S1). After applying a probe-abundance threshold of ≥0.001%, there was only 1 MAG represented by >10 probes for both the Allegro and JAX designs in the E. coli sample and 3 and 2 MAGs for the Allegro and JAX designs, respectively, in the Mock sample as expected (colored dots in Fig. 2a). For the E. coli sample, 99.95% and 99.28% of reads mapped to the E. coli genome for the Allegro and JAX designs, respectively. For the Mock community sample, 99.92% and 98.36% of reads mapped to the three genomes present in the Allegro design and two in the JAX design, respectively.

10.1128/mSphere.01325-20.1 FIG S1 Establishing thresholds. Thresholds for declaring a MAG present in a sample were determined using a no-template control (NTC), Escherichia coli genomic DNA, and ZymoBIOMICS Microbial Community Standard. Multiple probe-abundance thresholds were applied to the control samples (*x* axis). The number of MAGs with ≥10 probes at each minimum probe-abundance threshold is shown on the *y* axis. A probe abundance threshold of 0.001% improves accurate detection of MAGs. Download FIG S1, PDF file, 0.1 MB. Copyright © 2021 Benjamino et al. 2021 Benjamino et al. https://creativecommons.org/licenses/by/4.0/ This content is distributed under the terms of the Creative Commons Attribution 4.0 International license.

In negative-control samples, only a few thousand reads were obtained. NTC reads corresponded to 179 and 312 different probes and 77 and 138 MAGs in the Allegro and JAX designs, respectively (Fig. 2a). Of these probes, 94 (Allegro) and 142 (JAX) from E. coli overlapped the NTC probes and 66 (Allegro) and 96 (JAX) from the Mock overlapped the probes in the NTC. There are several potential sources of these reads: (i) contamination of the NTC with mouse stool DNA that was processed on the same batch, (ii) contamination of the reagents used for library preparation, or (iii) barcode-hopping during the sequencer run. Although many MAGs were matched by NTC reads, most of those MAGs were represented by only a few probes. No MAGs in the Allegro design and only one MAG in the JAX design had more than 10 probes represented in the NTC (Fig. 2a). The MAG with >10 probes in the JAX data set (single-China_7-4_110307.52) is a *Muribaculaceae* member and present at high abundance in many of the mouse samples.

The Allegro and JAX probe sets have no sequence overlap; thus, they represent two completely independent assays for relative abundance of hqMAGs in mouse specimens. High concordance in probe representation and relative abundance would therefore support both the reliability of the MA-GenTA assay and the structural validity of the detected MAGs as representing a species present in the test sample. The Allegro and JAX probe sets were used to assay DNA extracted from 72 mouse stool pellet samples, averaging 3.7 million sequencing reads per sample (Table 1; Table S1). All reads for both data sets were mapped to the iMGMC hqMAG reference. The two probe sets yielded similar numbers of sequencing reads and mapped reads (Table S1). There was a larger variation in the proportion of uniquely mapped reads and fewer on-target reads in the Allegro data set compared to the JAX data set, suggesting that the JAX probe design pipeline may be more effective in selecting unique regions of each MAG.

**TABLE 1:** Mouse specimen groups used for analysis

Study code	Summary	No. of samples	Data type	Reference	BioProject accession no.
HLB	C57BL/6J and HLB444 mice on chow and high-fat diet	29	16S	Svenson et al. (40)	PRJNA505515
			mWGS	Unpublished	PRJNA646227
			MA-GenTA	This study	PRJNA646241

CCF	C57BL/6J, CAST, and PWK mice	40	mWGS	J. Oh, W. Zhou, M. Adams, and J. Graham, unpublished	PRJNA646095
			MA-GenTA	This study	PRJNA646241

Comparison of the MAG abundances between the two designs gave a Pearson correlation coefficient of 0.98, demonstrating that the MAG abundances as measured by the Allegro and JAX probe sets were highly consistent (Fig. 2b). The points on the plot are colored by the number of probes detected in each MAG in both probe sets, showing higher abundance and better concordance between the probe sets for MAGs with reads from 10 or more probes. The MAGs were also plotted based on the number of probes detected in each data set across all mouse samples, illustrating that MAGs tend to have high or low probe representation in both probe sets (Fig. 2c). Using the thresholds described above to consider a MAG present in a sample if it had least 10 probes with ≥0.001% abundance reduced the number of MAGs being detected (Fig. 2d); however, these MAGs captured >90% of reads, suggesting that the MAGs below the thresholds are low-abundance genomes or noise (Fig. 2e).

### Comparison of the MA-GenTA assay to other microbial community profiling assays.

**FIG 3:**
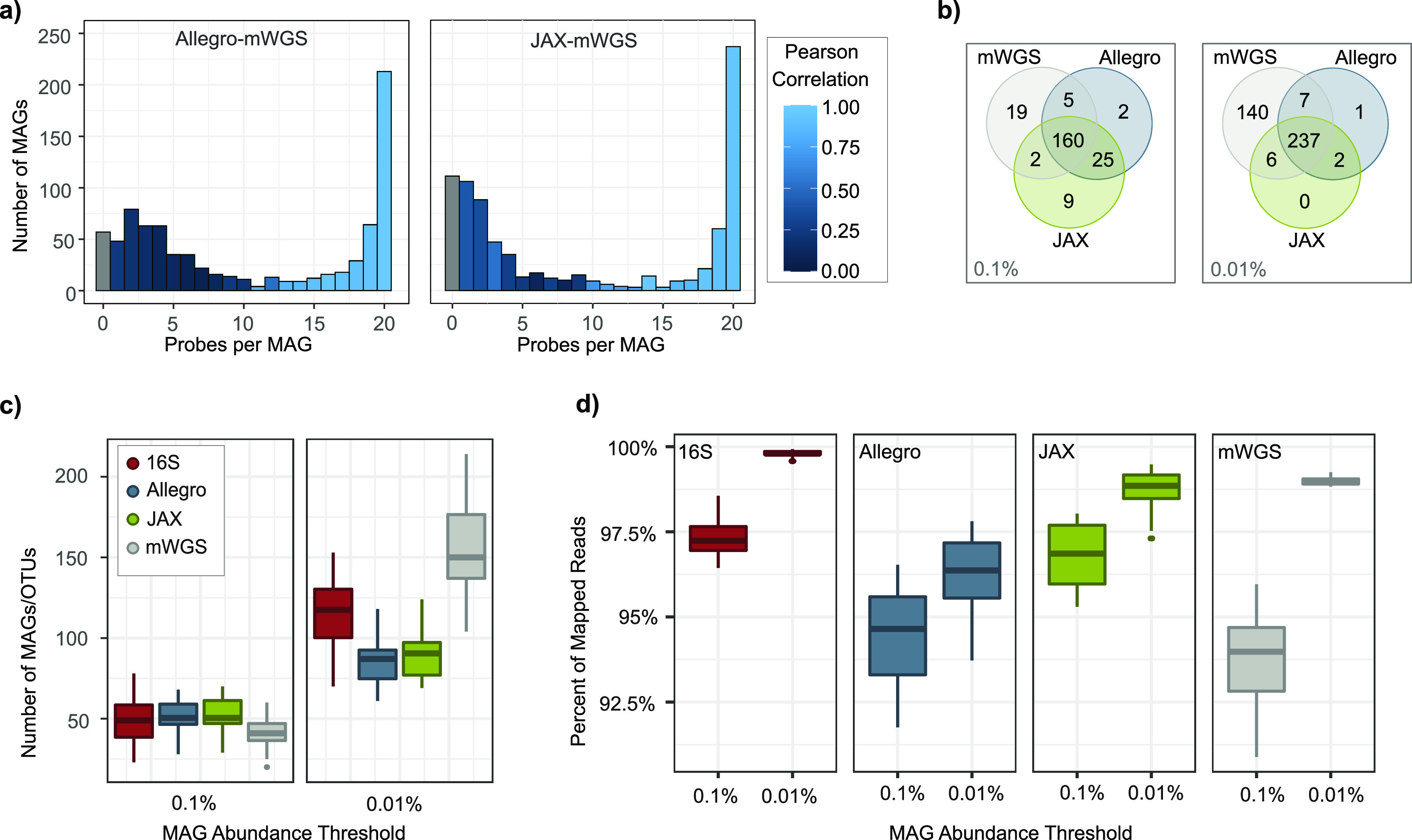
Comparison of MA-GenTA results with 16S OTU and mWGS analysis of mouse specimens. (a) The relative abundances of each MAG as inferred from the targeted and mWGS data were compared across the mouse stool samples using histograms showing the number of MAGs (*y* axis) with the number of probes observed per MAG (*x* axis) with no minimum probe-abundance threshold. The color scale shows the Pearson correlation of the relative abundance between the Allegro (left) and JAX (right) data and the mWGS data. (b) The total numbers of MAGs present in each assay (JAX, Allegro, and mWGS) are shown in Venn diagrams, highlighting the MAGs overlapping between the assays. (c) Samples from the HLB data set are shown with 16S rRNA v1-v3 OTUs and hqMAGs detected by Allegro, JAX, and mWGS assays at a range of minimum MAG/OTU-abundance thresholds. (d) The percentage of mapped reads for each assay at a range of minimum MAG/OTU-abundance thresholds.

mWGS data were available for 69 mouse stool samples, enabling correlation of relative abundance data for each MAG between the two assays. MAGs were separated into groups based on the number of probes (e.g., from 1 to 20) with at least 0.001% abundance in the MA-GenTA data for each sample, and a Pearson correlation was performed on each group of MAGs between the MA-GenTA and mWGS abundance data (Fig. 3a, Fig. S2 to S4, and Table S2). For both the Allegro and JAX data sets, MAGs with ≥15 probes detected have relative abundance correlations of *R* > 0.85 to the mWGS data. MAGs represented by fewer than 10 probes had poor Pearson correlations between the relative abundance of MA-GenTA and mWGS data (*R* ≤ 0.23 for Allegro and *R* ≤ 0.52 for JAX). Poor correlation between the data sets for MAGs with fewer probes could be due to poor probe performance, improperly assembled MAGs, pangenome differences between the MAG and the organisms present in our samples, or inflated abundance values in mWGS caused by spurious matches in conserved regions.

10.1128/mSphere.01325-20.2 FIG S2 Correlation of relative abundance between Allegro targeted data and mWGS data. MAG abundance data were separated into groups based on the number of probes per MAG (plot titles in gray). The abundance of each MAG in the group was compared to the MAG abundance determined by mWGS by Pearson correlations. The correlation values for each group are at the top of each plot. Download FIG S2, PDF file, 0.3 MB. Copyright © 2021 Benjamino et al. 2021 Benjamino et al. https://creativecommons.org/licenses/by/4.0/ This content is distributed under the terms of the Creative Commons Attribution 4.0 International license.

10.1128/mSphere.01325-20.3 FIG S3 Correlation of relative abundance between JAX targeted data and mWGS data. MAG abundance data were separated into groups based on the number of probes per MAG (plot titles in gray). The abundance of each MAG in the group was compared to the MAG abundance determined by mWGS data by Pearson correlations. The correlation values for each group are at the top of each plot. Download FIG S3, PDF file, 0.3 MB. Copyright © 2021 Benjamino et al. 2021 Benjamino et al. https://creativecommons.org/licenses/by/4.0/ This content is distributed under the terms of the Creative Commons Attribution 4.0 International license.

10.1128/mSphere.01325-20.4 FIG S4 Comparison of MAG abundance between MA-GenTA and mWGS. Number of MAGs (*y* axis) were plotted against the number of probes per MAG (*x* axis) for the Allegro and JAX data. Each MAG was colored by the percent abundance inferred by MA-GenTA and mWGS sequencing. Note that these graphs are the same as those shown in Fig. 3c but colored by relative abundance rather than Pearson correlation. Download FIG S4, PDF file, 0.2 MB. Copyright © 2021 Benjamino et al. 2021 Benjamino et al. https://creativecommons.org/licenses/by/4.0/ This content is distributed under the terms of the Creative Commons Attribution 4.0 International license.

10.1128/mSphere.01325-20.7 TABLE S2 Pearson correlation of MA-GenTA versus mWGS. Download Table S2, XLSX file, 0.01 MB. Copyright © 2021 Benjamino et al. 2021 Benjamino et al. https://creativecommons.org/licenses/by/4.0/ This content is distributed under the terms of the Creative Commons Attribution 4.0 International license.

We next compared the number of observed MAGs from the MA-GenTA assay with the number of 16S rRNA v1-v3 operational taxonomic units (OTUs) and MAGs detected in the mWGS data across the mouse samples from the HLB study. The sensitivity to detect a MAG in the MA-GenTA data depends upon sequencing depth (more reads means it is more likely that reads from a low-abundance genome will be detected) and probe representation (if a MAG truly represents the genome of a species present in the sample, then reads from a large fraction of probes should be observed). A MAG was considered present in the MA-GenTA data if at least 10 probes had >0.001% probe abundance. These thresholds were used in subsequent analyses of mouse stool data sets.

Venn diagrams for MAG abundance thresholds of 0.1% and 0.01% show high overlap of MAGs detected between JAX and Allegro MA-GenTA data sets, with an increasing number of low-abundance MAGs detected only in the mWGS assay (Fig. 3b). The numbers of observed OTUs and MAGs per sample in the 16S, mWGS, and MA-GenTA data sets were determined based on OTU and MAG relative abundance thresholds of 0.1% and 0.01% (Fig. 3c). The two MA-GenTA assays were consistent at both thresholds and similar to the number of OTUs at the 0.1% threshold. The number of OTUs and MAGs in 16S and mWGS data were higher than those from the MA-GenTA assays at the 0.01% threshold. 16S OTUs are not reference dependent and therefore capture a larger fraction of the bacterial species than MAGs do, albeit without the same level of phylogenetic specificity. The lower number of MAGs seen in the MA-GenTA data may also be attributed to the probe abundance (0.001%) and probes per MAG (10 ppM) thresholds previously applied to ensure high confidence of MAG presence, whereas no such thresholds are applied to 16S or mWGS data. While the mWGS samples had more MAGs present at ≥0.01% relative abundance (Fig. 3c), there was only a 4% average increase in mapped reads per sample (Fig. 3d). The percentage of reads in the JAX data set mapped to MAGs with ≥0.1% abundance was, on average, similar to the percentage of reads mapped in the mWGS data set.

**FIG 4:**
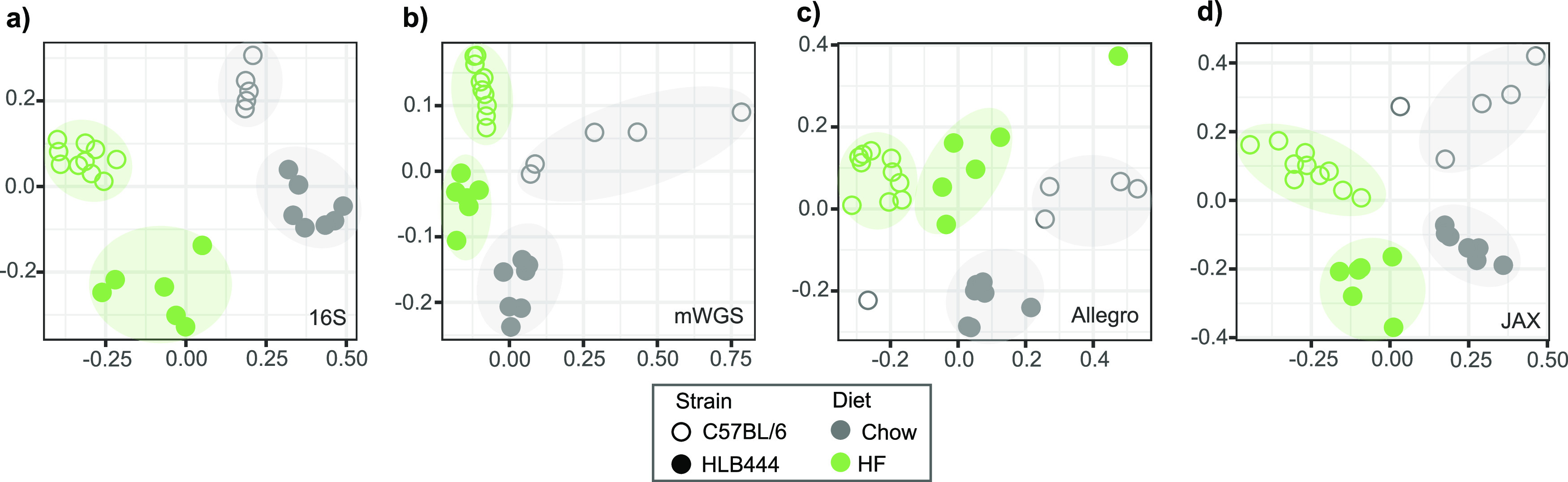
MA-GenTA as an assay for experimental group differentiation and functional analysis. (a) The Bray-Curtis dissimilarity metric was applied to HLB data from each sequencing assay and shown in nonmetric multidimensional scaling (NMDS) plots. Points are colored by diet, closed circles represent HLB444 samples, and open circles are C57BL/6J samples. All four sequencing assays cluster points based on diet and mouse strain.

In order to demonstrate the utility of the MA-GenTA assay in characterizing microbial profiles in an experimental context, we used the MA-GenTA data sets for analysis of the HLB samples. Prior results identified OTU differences between C57BL/6J mice and HLB444 mice, which carry a mutation in the *Klf15* gene, on both a standard chow diet and after introduction of a high-fat, high-sugar diet (HF). HLB444 mice were resistant to diet-induced obesity when fed the HF diet. To determine the ability of the MA-GenTA assay to differentiate these groups, the Bray-Curtis dissimilarity metric was applied to the 16S, mWGS, and MA-GenTA data of the same samples and viewed with nonmetric multidimensional scaling (NMDS) plots (Fig. 4). All assays showed samples clustered by diet (Chow versus HF) and mouse strain (C57BL/6J versus HLB444). Permutational multivariate analysis of variance (PERMANOVA) for each of the sequencing assays confirmed significant clustering between mouse strain and diet: Allegro assay (*f* = 2.6961, *P* = 0.0029), JAX assay (*f* = 13.629, *P* = 0.0009), 16S (*f *= 19.581, *P* = 0.0009), and mWGS (*f* = 2.05, *P* = 0.0099) (Table S3). Overall, there were few differences in the distribution of Bray-Curtis dissimilarity values as determined by all four methods: 16S, JAX and Allegro MA-GenTA and mWGS (Fig. S5).

10.1128/mSphere.01325-20.5 FIG S5 Bray-Curtis dissimilarity comparisons between analysis methods. The Bray-Curtis dissimilarities between samples were calculated for each analysis method (16S, mWGS, Allegro, and JAX). A Kruskal-Wallis test followed by Dunn’s multiple-comparison test was performed for each design per sample comparison. Comparisons denoted with an asterisk are significantly different (*P* < 0.05). Download FIG S5, PDF file, 0.8 MB. Copyright © 2021 Benjamino et al. 2021 Benjamino et al. https://creativecommons.org/licenses/by/4.0/ This content is distributed under the terms of the Creative Commons Attribution 4.0 International license.

10.1128/mSphere.01325-20.8 TABLE S3 PERMANOVA statistics of Bray-Curtis dissimilarity in different sequencing assays. Download Table S3, XLSX file, 0.01 MB. Copyright © 2021 Benjamino et al. 2021 Benjamino et al. https://creativecommons.org/licenses/by/4.0/ This content is distributed under the terms of the Creative Commons Attribution 4.0 International license.

### Functional analysis using MA-GenTA.

**FIG 5:**
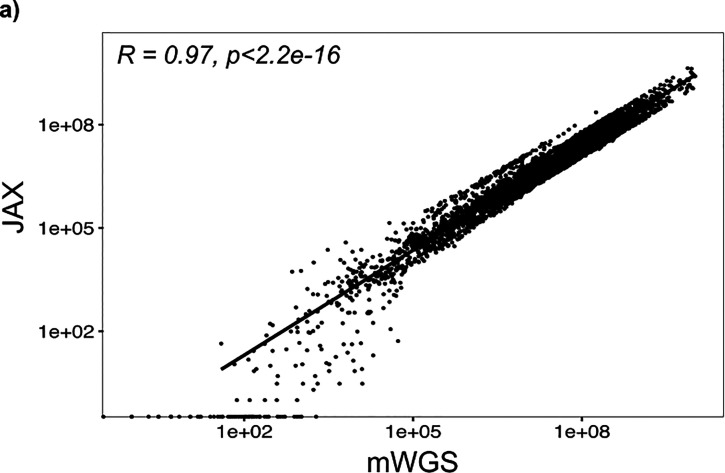

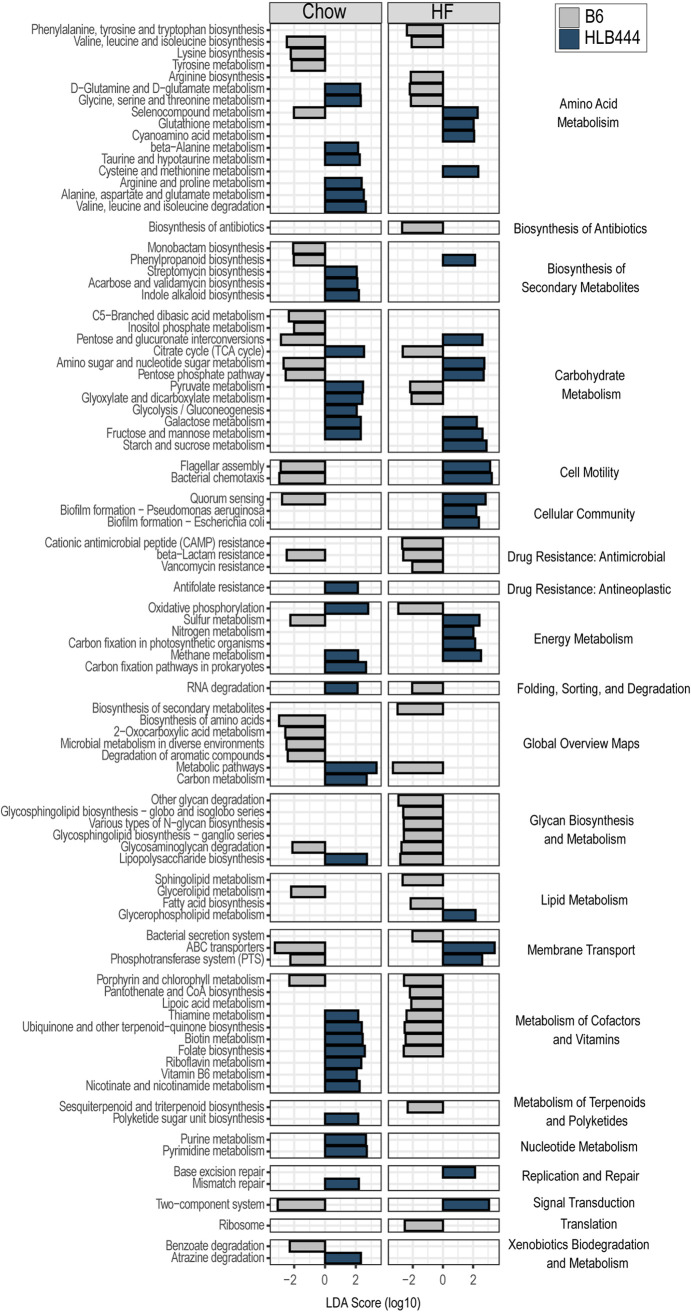
Functional pathway analyses with LEfSe. (a) A Pearson correlation of pathway abundance for each sample as inferred by mWGS and MA-GenTA (JAX) demonstrates the similarity of KEGG pathway assignments between the two assays. (b) LDA of KEGG pathways inferred by MA-GenTA (JAX) MAG abundances shows differentially abundant pathways between HLB444 and B6 mouse strains on chow and HF diets.

Given the relative abundance of MAGs in each sample, we inferred the functional potential of each sample based on annotation of proteins encoded in each MAG to KEGG pathways. MA-GenTA and mWGS read counts for each MAG in the HLB samples were assigned to KEGG pathways on a per-sample basis, and the relative abundance was determined for each pathway. A Pearson correlation was used to compare the read counts of each KEGG pathway per sample between mWGS and MA-GenTA data sets (Fig. 5a). The results between the two data sets were highly correlated. The mWGS sequences were also analyzed using HUMAnN2; however, only ∼1% of the reads could be assigned to pathways, markedly limiting the utility of the results (data not shown). Linear discriminant analysis (LDA) in LDA effect size (LEfSe) was used to determine differentially abundant pathways between the two mouse strains and the two diets from the HLB study. The number of differentially abundant pathways (MA-GenTA) varied across comparisons (HLB444 versus B6 on HF diet [53 and 60], HLB444 versus B6 on Chow [63 and 66], Chow versus HF in HLB444 [101 and 103], and Chow versus HF in B6 [75 and 81]) for the Allegro and JAX data sets, respectively (Table S4). All LEfSe results for the Allegro and JAX designs are shown in Table S4, and the JAX data are highlighted in Fig. 5b. Concordance of differentially abundant KEGG pathways between the two data sets was 82% for HLB444 versus B6 on HF, 72% for HLB444 versus B6 on Chow, 96% for Chow versus HF in HLB444, and 77% for Chow versus HF in B6. Consideration of the response of HLB444 and B6 strains to the HF diet showed differences in carbohydrate metabolism between the two strains on the HF diet, with HLB444 animals having higher representation of glycolysis, tricarboxylic acid (TCA) cycle, and oxidative phosphorylation and B6 animals having higher representation of pathways related to utilization of other sugars (Fig. 5b). These and other differences distinguished the response to HF diet of these two mouse strains and suggest that microbial differences contribute to the ability of HLB mice to adapt to the HF diet.

10.1128/mSphere.01325-20.9 TABLE S4 LEfSe results for differentially abundant pathways. Download Table S4, XLSX file, 0.03 MB. Copyright © 2021 Benjamino et al. 2021 Benjamino et al. https://creativecommons.org/licenses/by/4.0/ This content is distributed under the terms of the Creative Commons Attribution 4.0 International license.

### Specificity of MA-GenTA in a complex microbial environment.

**FIG 6:**
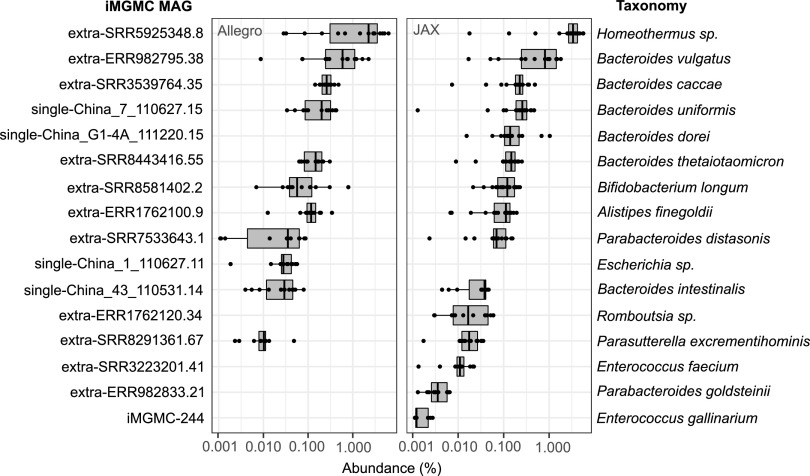
MA-GenTA as a precise assay. The Allegro and JAX probe pools were used on a human stool sample to test the detection reliability of the probes in a complex sample. MAGs shown have at least 10 probes present at ≥0.001% relative abundance.

As an additional way to assess the specificity of probe targeting, both probe sets were used to assay metagenomic DNA extracted from a human stool specimen, which serves as a highly complex microbial sample with few organisms in common with mouse gut bacteria (Fig. 6). While there are deep-branching similarities in the gut microbiota of human and mouse, there are major differences at the genus and species level (11, 19, 20). There were 16 MAGs detected in the human stool sample using the same thresholds for detection as used for the mouse samples (minimum of 10 probes per MAG at ≥0.001% probe abundance). The taxa associated with the detected MAGs have previously been found in human stool samples (21–27).

## DISCUSSION

As the field of microbial community profiling grows, the need for informative, cost-effective, and streamlined assays of microbial composition becomes more important. Although it was initially developed for genotyping applications, we have shown that by combining results from multiple rigorously selected probes per genome, the Allegro Targeted Genotyping assay can produce accurate microbial relative abundance data across a dynamic range of at least 3 orders of magnitude at a cost that is only moderately higher than 16S rRNA profiling. MA-GenTA bridges the gap between two commonly used approaches—16S rRNA gene sequencing and mWGS—combining some of the strengths of each (Table 2). Other approaches have been described that attempt to balance cost with informativeness, including metagenomic multilocus sequence typing (MLST) (56), full-length 16S rRNA profiling (57, 58), and shallow-coverage mWGS targeting as few as 0.5 million reads/sample (59).

**TABLE 2:** Comparison of microbial community profiling assays

Feature	16S rRNA gene sequencing	Whole metagenome sequencing	MA-GenTA
Taxonomic resolution	Approx family/genus level for 16S rRNA subregions; strain level for full-length gene	Species/strain level	Species/strain level

Gene content	None	High	Inferred based on genome matches

Analysis complexity	Medium	High	Medium

Cost	<$50/sample	>$100/sample	$50–$75/sample

Pros	Quick community survey	New organism/gene discovery	Efficient pooled-sample workflow
	Large no. of studies from many environments/hosts	Direct comparison of data sets with same reference for mapping	Customized target selection/pool composition
			Direct comparison of data sets with same reference for mapping

Cons	Limited taxonomic specificity	Possible misassignment of reads to closely related organisms	Limited to existing organisms/genomes
	No gene content information	Cost	Limited pangenome characterization

Capture-based targeted sequencing methods have been widely used for exome sequencing and cancer mutation profiling (3, 28, 60) and represent another potential alternative approach for targeted microbiome profiling. Guitor et al. recently described a method for highly multiplexed detection of antibiotic resistance genes and bacteria that relies on biotinylated capture probes (29, 30). These probes and streptavidin bead capture kits are costly and require each specimen to be processed separately, making library preparation laborious. In contrast, the Allegro workflow uses unmodified oligonucleotides; pooling after a sample-specific index-tagging step and combination of pools can yield up to 3,072 uniquely barcoded libraries on a single sequencing run. Cost comparisons necessarily incorporate many variables, but as performed here, the MA-GenTA assays cost about twice that of 16S rRNA sequencing. Unlike array-based platforms (31), it is straightforward to alter the design of the MA-GenTA probe pool with each reagent order, allowing both the refinement of the selected probes for each genome and the inclusion of additional content over time. Each approach has advantages and disadvantages. A discriminating feature of MA-GenTA is that the integration of information across multiple unique probes per genome may provide a more robust estimate of genome abundances.

Probe selection is an important factor in achieving genome specificity. In the JAX design, half of the probes for each MAG were designed to universal single-copy prokaryotic genes with a focus on aminoacyl-tRNA synthetases. We did not observe any difference in performance between probes that targeted these genes and other probes (data not shown). Targeting single-copy genes may provide additional advantages for species-tagging applications, although care must be taken to ensure probe specificity when using strongly conserved target genes. As the MA-GenTA assay depends on probe hybridization to DNA within a sample, an important factor to consider when designing a probe pool is the reference database from which probes are chosen, including how representative the database is of organisms present in the sample. While the iMGMC reference database is the most comprehensive to date for the mouse gut, only about 50% of the mouse mWGS reads used in this study had high-quality matches to the iMGMC hqMAGs, reinforcing the need for a more robust reference for the mouse stool microbial community.

A hallmark and major motivation of mWGS sequencing is the ability to analyze functional capability of the organisms in an environment. Strategies have been described to predict function based on OTU composition (32–34), but they are strongly dependent on the reference databases and perform poorly on data sets from non-human-associated microbes (35). Because probe design for the MA-GenTA assay requires reference genomes, this approach does not contribute to bacterial discovery. However, gene and pathway abundance data can be inferred from MA-GenTA data by pairing read counts to pathways represented in the reference genomes more directly than is possible based on 16S rRNA sequences and at lower cost than mWGS sequencing.

The ability to synthesize probes based on user-defined parameters allows for broad or targeted study of microbial communities, specific species or strains, genes of interest, and antibiotic resistance or virulence markers. Probe designs that focus on universal genes may be a good choice for species tagging, while probes targeting variable regions could provide additional information on pangenome variation. Further optimization of the MA-GenTA assay might involve adjusting the number of probes per genome and how thresholds for probe abundance and probe representation are used to reduce noise and increase confidence of MAG assignment. Although not examined here, the specificity of the MA-GenTA assay would also be advantageous in specimens with high proportions of host genomic DNA such as nasal and oral samples for targeting rare organisms in complex microbiomes where mWGS analysis is inefficient. The MA-GenTA assay could also be adapted to a transcriptome sequencing (RNA-seq) format for targeted gene expression analysis.

## MATERIALS AND METHODS

### Probe design and filtering.

The “high-quality” MAG set from the integrated Mouse Gut Metagenomic Catalog (iMGMC) was accessed from GitHub (https://github.com/tillrobin/iMGMC). The hqMAG set comprised 830 dereplicated genome equivalents predicted to be >90% complete and <5% contaminated based on analysis by CheckM (16). Two probe design strategies were used. For the JAX design, the probe selection program CATCH (36) was run on each hqMAG separately to design over 50,000 40-base probes per MAG. BLAST was used to match probes to Prokka-annotated open reading frames (ORFs) (37). Probes with BLAST matches shorter than 40 bp in length or less than 100% identity were removed, followed by probes corresponding to genome regions on a predefined discard list. Discard regions included annotations listed as tRNAs, ribosomal proteins, and those with encoded proteins with the term “repeat” or “hypothetical” in the name. Probes were required to have between 45 and 65% G+C nucleotides. Probes with multiple matches within the hqMAG or to more than one hqMAG were also excluded. Probes matching the single-copy MUSiCC gene list (38) were flagged for probe selection. All resulting probes were sent to Tecan Genomics (Redwood City, CA) where probe compatibility was assessed for probe pool production based on the Allegro Targeted Genotyping protocol, and probe pools with 20 probes per MAG were synthesized (JAX design), with 10 representing MUSiCC genes and 10 representing non-MUSiCC genes. The iMGMC hqMAGs were also used by Tecan Genomics to create a second probe pool (Allegro design) with 20 probes per MAG. There were 16 MAGs that did not pass probe-synthesis filtering metrics for the JAX design but were present in the Allegro design. The final probe pools contained 16,600 probes for the Allegro design and 16,280 probes for the JAX design. Cross-reference between the hqMAG set and the ZymoBIOMICs Microbial Community Standard was determined using BLAST alignment (39), resulting in 3 MAGs matching genomes from the ZymoBIOMICS genomes (Escherichia coli, Enterococcus faecalis, and Pseudomonas aeruginosa).

### DNA Extraction of mouse stool pellets and controls.

Genomic DNA isolated from mouse stool pellets from several studies was used for evaluation of the MA-GenTA assay (Table 1). All procedures used for animal husbandry and collection of specimens were approved by the Jackson Laboratory Animal Care and Use Committee, and research was conducted in conformity with the Public Health Service Policy on Humane Care and Use of Laboratory Animals. The HLB study pellets and positive controls (E. coli, ZymoBIOMICS Mock) were lysed using Qiagen PowerBead garnet tubes with 1 ml Qiagen InhibitEX buffer. The lysate was then processed with the QIAcube HT instrument using a modified Qiagen QIAamp 96 DNA QIAcube HT protocol (40). Each sample (a single stool pellet, 10 to 60 mg total weight) was added to a Qiagen PowerBead 0.7-mm garnet tube with 1 ml of Qiagen InhibitEX buffer. All samples were incubated at 65°C for 10 min followed by 95°C for 10 min. The samples were then mechanically lysed for 2 cycles of 30 s at 3,700 rpm on a Qiagen PowerLyzer 24 homogenizer, with a 1-min rest period between cycles. Samples were then centrifuged at 10,000 × *g* for 1 min, and then 200 μl of this lysate was then mixed with AL buffer (285 μl) and proteinase K (5 μl). The lysate was incubated for 10 min at 70°C, followed by an ice incubation for 5 min. Four hundred eighty-five microliters of lysate was transferred to a QIAcube HT instrument, where the lysate was combined with 200 μl of 100% ethanol and then bound to the QIAamp 96 plate. Each well of the QIAamp 96 plate was then washed with 600 μl of AW1 buffer and AW2 buffer and then 100% ethanol. DNA was then eluted with 100 μl of AE buffer without using TopElute fluid. The collaborative cross founder (CCF) stool pellets were homogenized with 500 μl tissue and cell lysis buffer (Lucigen) by pipetting up and down. An aliquot of 100 μl was removed and treated with an enzyme cocktail (5 μl 10-mg/ml lysozyme, 1 μl lysostaphin [5,000 U/ml], 1 μl mutanolysin [5,000 U/ml], and 20 μl tissue and cell lysis buffer) for 30 min at 37°C. Buffer ASL (Qiagen) (200 μl with 0.5 μl antifoaming agent DX) was added to each tube and mixed. Samples were placed on a Qiagen TissueLyser II bead beater for 2 periods of 3 min each (30 Hz) and then spun down in a microcentrifuge. Each sample (200 μl) was further processed on the Qiagen QIAamp 96 DNA QIAcube HT protocol. The same isolated DNA preparation from each specimen was used for collection of 16S OTU, mWGS, and MA-GenTA data.

### Allegro Targeted Genotyping sample preparation and sequencing.

The Allegro Targeted Genotyping V2 protocol (publication number M01501; Tecan Genomics, Inc.) was followed for library preparation of all samples in duplicate with the Allegro and JAX probe pools. Briefly, gDNA samples were enzymatically fragmented, followed by ligation of barcoded adaptors. Barcoded samples were then purified and pooled in groups of 48. Each pool of 48 samples was placed in an overnight annealing and extension reaction mixture with the probe pool, followed by an AMPure XP bead purification. A qPCR step was used to determine the number of cycles used in the library amplification (18 cycles). Amplified libraries were bead purified (AMPure XP) and pooled in equimolar ratios for sequencing. A no-template control (NTC), Escherichia coli gDNA (ATCC 8739), a human stool metagenome DNA sample (41), and a defined composition microbial community control (ZymoBIOMICS Microbial Community Standard, catalog no. D6300) were used as controls. Libraries created from the Allegro Targeted Genotyping assay were pooled and sequenced on an Illumina NovaSeq SP 2- by 150-bp run, using the custom R1 primer and 1% spike-in of the phiX174 library as recommended. Libraries were loaded on the NovaSeq SP at 60% of standard loading per Allegro Targeted Genotyping assay recommendation; only forward read data were used for analysis.

### Data analysis. (i) mWGS read mapping and 16S OTU generation.

mWGS libraries were prepared using Illumina NexteraXT kits, and 2- by 150-base reads were obtained on the HiSeq NovaSeq. The raw mWGS sequences were trimmed of adaptors and low-quality bases using Cutadapt version 1.14 (42). Host contaminant sequences were identified and filtered out using Kraken2 version 2.0.8-beta (43). The clean sequences were aligned against the reference (iMGMC MAGs) using BWA version 0.7.12 (44) with parameter settings: bwa mem -M -P. The nonprimary alignment reads were then filtered out using SAMtools version 0.1.19 (45) with parameter setting -F 256. Reads were filtered using 97.5% identity and 50% coverage thresholds. Finally, the read count table by bin for each sample was generated from the alignment file. On average, about 50% of total mWGS reads mapped to the iMGMC 830 hqMAGs. 16S OTUs were generated for the HLB data with USEARCH, using previously published parameters (40, 46).

### (ii) MA-GenTA read mapping and data analysis.

Raw sequences were trimmed using TrimGalore/Cutadapt to remove the 40-bp probe (https://github.com/FelixKrueger/TrimGalore) (42). Read mapping to hqMAGs was performed using BWA. Sequences of up to 110 bp downstream of the probes were mapped to the iMGMC reference index. Reads mapped with <95.5% identity and ≤50% query length were removed. Secondary alignments with lower alignment scores were removed, and then reads mapped to multiple sites with similar alignment scores were removed, which resulted in uniquely mapped reads. BEDTools intersect command was used to match read alignment locations to the genome locations of the designed probes to provide “on-target” read counts, removing reads that aligned to regions outside the expected probe annealing location (47). Count tables were created representing the on-target read count and relative abundance of each probe in each hqMAG, and the summed read counts and relative abundance for all probes per hqMAG were used for analyses. All analyses were performed in R (version 4.0.2) (48). Allegro and JAX designs were compared based on the relative abundance per MAG and the number of probes per MAG matched in each sample. A Pearson correlation was performed on the MAG abundance comparison between the two designs and between each design and the relative abundance based on mWGS sequencing. The JAX and Allegro data were compared to 16S and mWGS data for the same samples on the basis of alpha (observed) and beta diversity (Bray-Curtis dissimilarity) metrics using Phyloseq (49).

### (iii) Functional analysis.

Protein coding sequences in the hqMAGs were predicted using Prodigal (50), implemented in Prokka (37). Functional annotation of the predicted coding DNA sequence (CDS) regions was performed by eggNOG-Mapper (51), using Diamond (52) for searches, and with overlap parameters requiring at least 25% query and reference coverage. For each sample, the number of reads mapping to each MAG was assigned to each KEGG pathway (53) for all constituent CDS regions. Differences in pathway abundance among sample groups were determined using linear discriminant analysis effect size with LEfSe (54). The mWGS data were analyzed using HUMAnN2 using default settings with Diamond as the protein-search method (55).

### Code availability.

All code used for probe design and data analysis, along with read count tables, has been deposited to GitHub at https://github.com/TheJacksonLaboratory/MA-GenTA.

### Data availability.

Sequence data created in this study have been deposited in GenBank with the BioProject accession no. PRJNA646241. The probe sequences used for this study have been deposited to GitHub at https://github.com/TheJacksonLaboratory/MA-GenTA.
