# Supplementary figures and images for: Genome-Based Targeted Sequencing as a Reproducible Microbial Community Profiling Assay
Source: mSphere. 2021 Apr 7;6(2):e01325-20. doi: 10.1128/mSphere.01325-20 (PMC8546724; doi:10.1128/mSphere.01325-20)

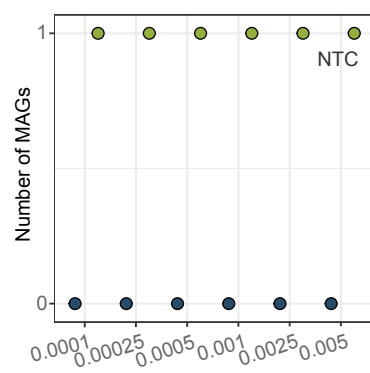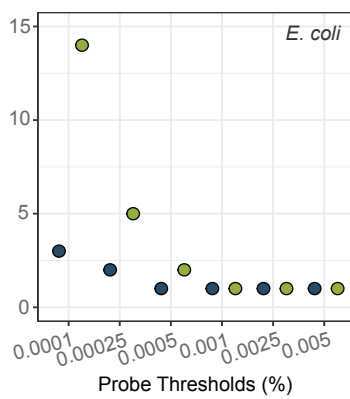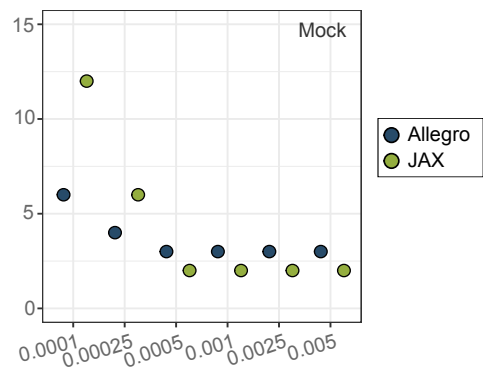

Supplement: FIG S1 [file msphere.01325-20-sf001.pdf]

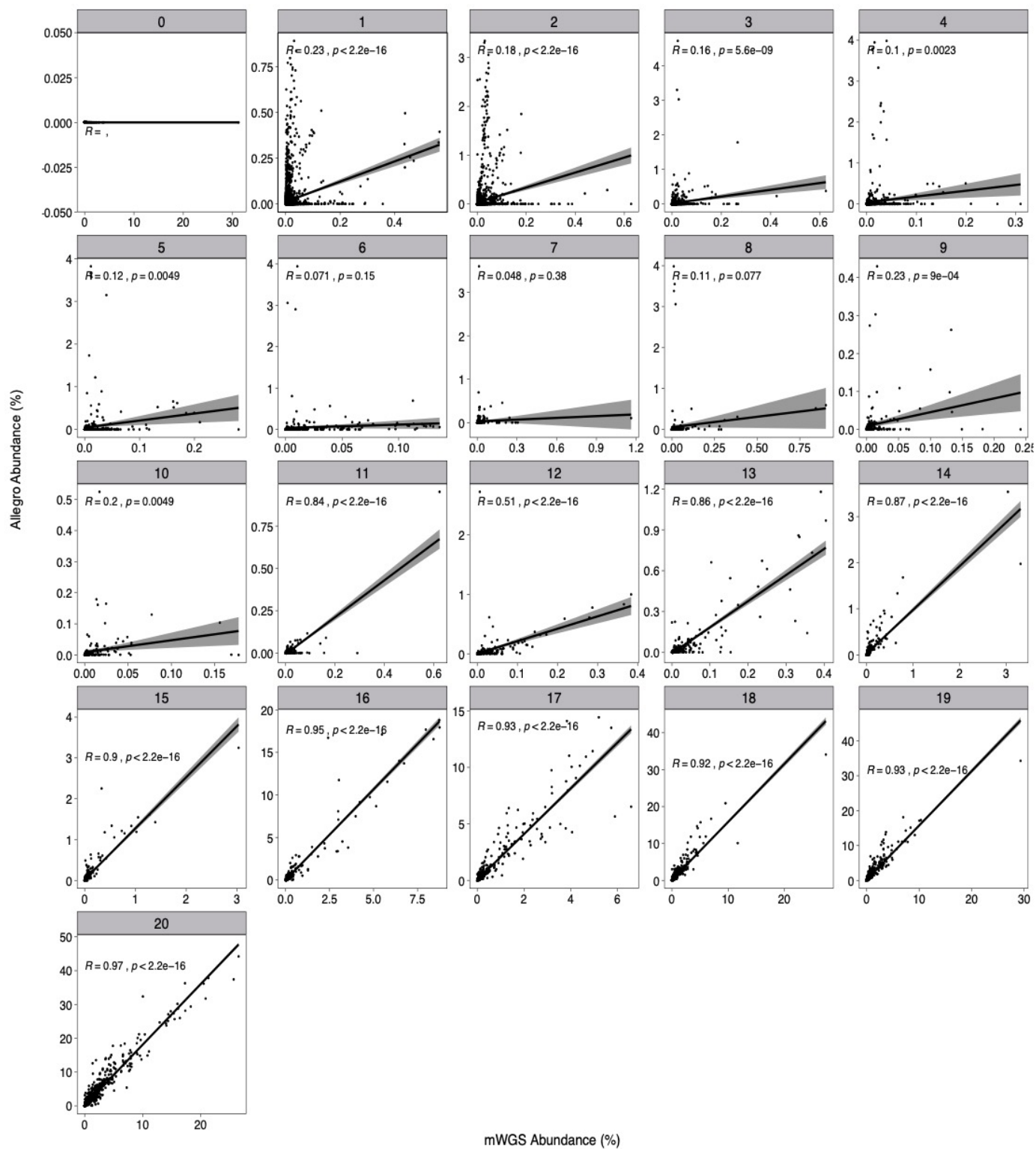

Supplement: FIG S2 [file msphere.01325-20-sf002.pdf]

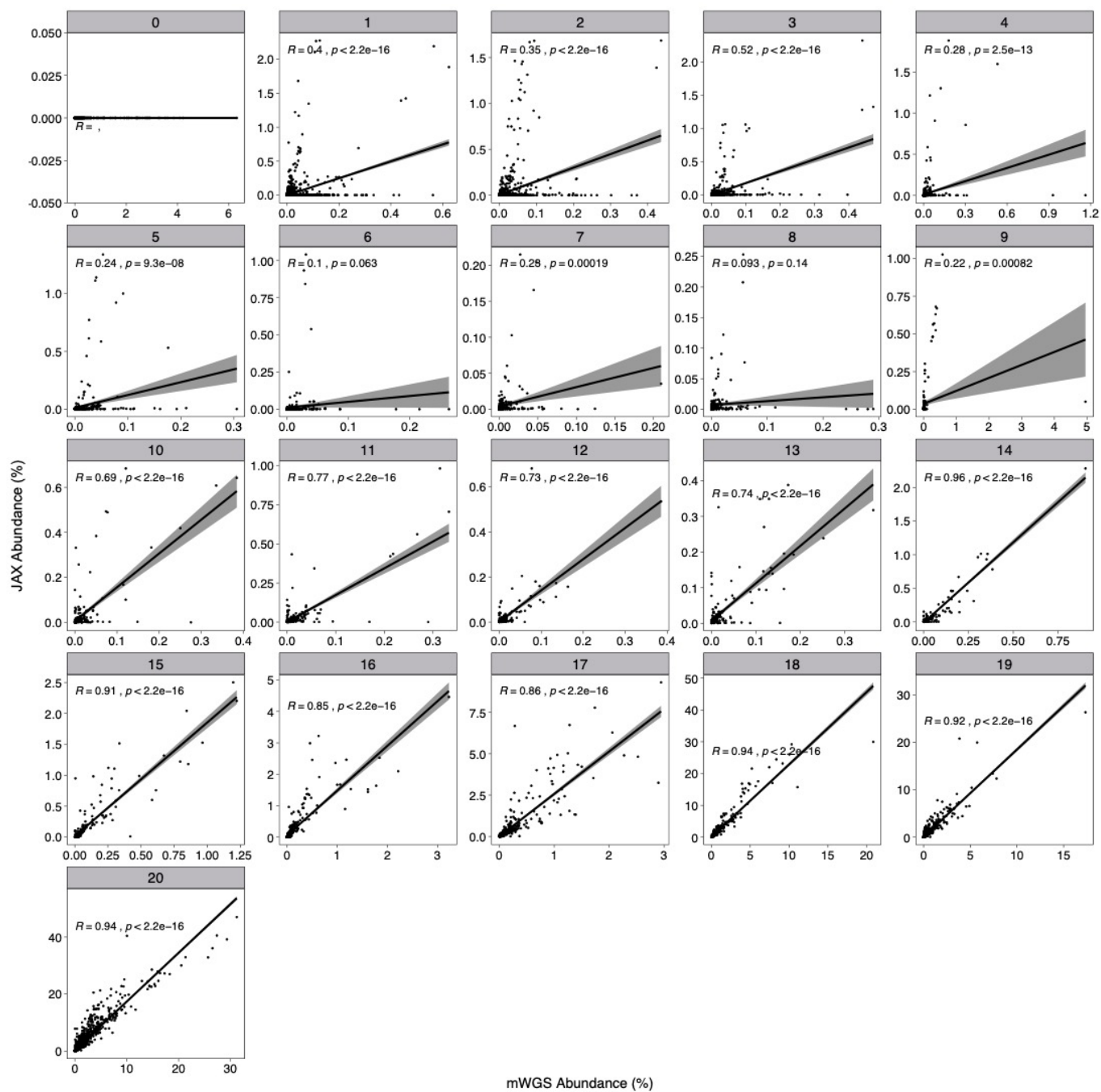

Supplement: FIG S3 [file msphere.01325-20-sf003.pdf]

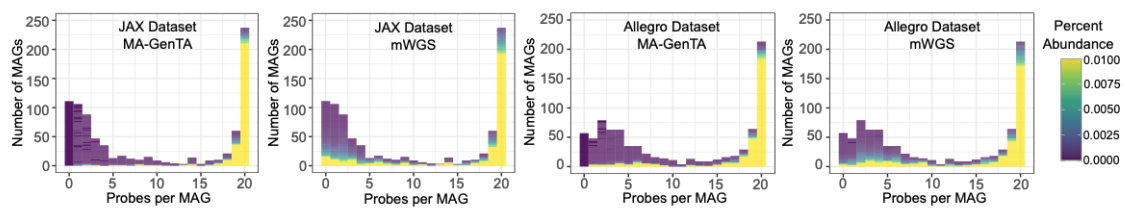

Supplement: FIG S4 [file msphere.01325-20-sf004.pdf]

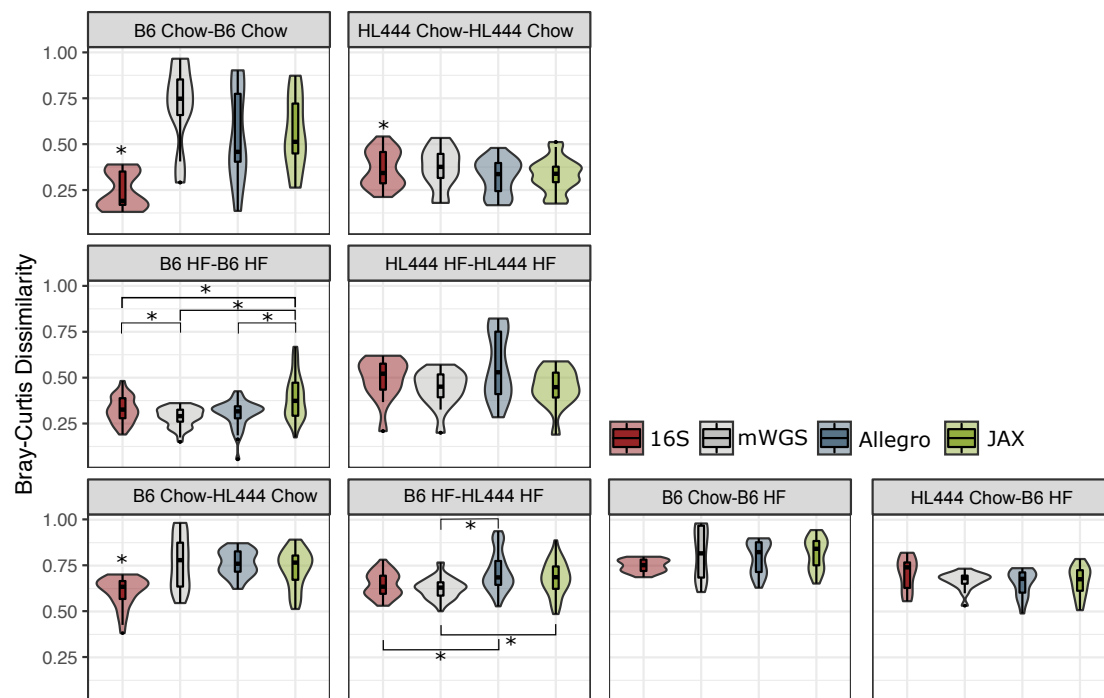

Supplement: FIG S5 [file msphere.01325-20-sf005.pdf]
